# Exploiting Epigenetic Alterations in Prostate Cancer

**DOI:** 10.3390/ijms18051017

**Published:** 2017-05-09

**Authors:** Simon J. Baumgart, Bernard Haendler

**Affiliations:** Drug Discovery, Bayer AG, Müllerstr. 178, 13353 Berlin, Germany; simon.baumgart@bayer.com

**Keywords:** androgen receptor, epigenetics, histone, prostate cancer, microRNA

## Abstract

Prostate cancer affects an increasing number of men worldwide and is a leading cause of cancer-associated deaths. Beside genetic mutations, many epigenetic alterations including DNA and histone modifications have been identified in clinical prostate tumor samples. They have been linked to aberrant activity of enzymes and reader proteins involved in these epigenetic processes, leading to the search for dedicated inhibitory compounds. In the wake of encouraging anti-tumor efficacy results in preclinical models, epigenetic modulators addressing different targets are now being tested in prostate cancer patients. In addition, the assessment of microRNAs as stratification biomarkers, and early clinical trials evaluating suppressor microRNAs as potential prostate cancer treatment are being discussed.

## 1. Introduction

Prostate adenocarcinoma is one of the most frequent male malignancies in developed countries and is a leading cause of cancer-related deaths. Its increasing rate is linked in part to the overall ageing of the population, particularly in the Western world [1]. It remains indolent in most cases but can later advance to aggressive prostate cancer. Despite initial good response to androgen deprivation therapy, most men with metastatic disease will eventually progress to castration-resistant prostate cancer (CRPC), where the androgen receptor (AR) axis still plays an essential role [2,3,4].

The disease is characterized by a high genetic heterogeneity which results in variable progression rates and difficult choices when it comes to treatment [3,4,5,6]. This heterogeneity is due to the multifocal origin of the disease [7] and the incremental accumulation of mutations during tumor progression. This was evidenced by extensive genomic profiling analyses performed on primary tumors [8] and on metastatic samples [2]. Importantly, these studies confirmed the essential role of the AR axis in early and late stages of the disease, thus vindicating ongoing efforts towards the identification of more efficacious AR antagonists and androgen synthesis inhibitors [9,10]. They furthermore show that genetic alterations with impact on the phosphatidylinositol-4,5-bisphosphate 3-kinase (PI3K) pathway and the DNA repair process are overrepresented [5,11], and indeed a number of studies document that these pathways interact with AR signaling [12,13]. Nonetheless, the precise molecular mechanisms involved in the progression of prostate cancer are still insufficiently understood [14,15,16].

Beside genetic mutations, diverse epigenetic changes are also likely to significantly contribute to prostate cancer progression [17,18,19]. Importantly, signaling downstream of AR activation is closely regulated by epigenetic modifications, suggesting that interfering with local chromatin modulation may represent a promising novel strategy to block androgen-mediated gene transcription. This area has attracted much attention due to the anticipated reversibility of epigenetic alterations and the recent identification of potent and selective inhibitors of epigenetic tumor drivers [20,21].

An epigenetic aberration which is often observed in prostate cancer is the global reduction of DNA methylation, but the existence of a causal link awaits further confirmation [22]. On the other hand, local DNA hypermethylation leading to the silencing of tumor suppressor genes occurs frequently [23]. In addition, multiple changes in the distribution of post-translational modifications (PTMs) of histones have been reported in prostate cancer. Increased and decreased histone acetylation levels have both been observed in prostate adenocarcinomas [24,25,26]. Modifiers and readers of histone acetylation were evaluated in detail for their oncogenic role in prostate cancer and potential targets for treatment identified [17,27]. Also, aberrant histone methylation, both at lysine and arginine residues, has been documented at different stages of prostate cancer, as well as expression changes and mutations in enzymes that add or remove these histone marks [17]. Finally, the investigation of microRNA dysregulation in prostate cancer is coming of age and accumulating data suggest that they may represent useful biomarkers for disease progression and treatment response [28]. Also, first studies with suppressor microRNAs show some anti-tumor efficacy in preclinical prostate cancer models [29].

## 2. Epigenetic Events in Prostate Cancer and Preclinical Efficacy of Inhibitors of Epigenetic Targets

### 2.1. DNA Methylation

DNA methylation is performed by dedicated DNA methyltransferases (DNMTs) that use S-adenosyl methionine as donor and predominantly modify CpG dinucleotides [23]. DNMT expression and activity are elevated in prostate tumor models [30], and also in androgen-resistant prostate cancer cell lines [31]. The expression of several genes, including *GSTP1* and *HOX* family members, is recurrently down-regulated in prostate cancer due to promoter hypermethylation [23]. This and other studies led to the proposal of panels of DNA methylation markers for the diagnosis of cancerous prostate tissue [32]. Interestingly, in CRPC, androgen target genes are more prone to changes in DNA methylation, in line with the continuous implication of the AR axis in late-stage disease [33]. Recently, a stratification of prostate cancer subtypes based on DNA methylation patterns has been proposed [8], but the clinical usefulness is still unclear.

The DNMT inhibitors azacitidine and decitabine have been evaluated in vivo in prostate cancer xenografts and showed some efficacy [34,35,36]. With the help of an improved formulation, a strong anti-tumor effect was observed for decitabine in two different prostate cancer xenografts [37]. Decitabine also prevents tumor growth in the transgenic adenocarcinoma of the mouse model [38].

One eraser of DNA methylation is the DNA hydroxymethylase ten–eleven translocation 1 which can revert cytosine methylation and was described as tumor suppressor [39]. Its expression is often reduced in prostate cancer tissue and associated with decreased survival [40].

### 2.2. Histone Acetylation

Global and local acetylation results from the balanced activity of cellular histone acetyltransferases (HATs) and histone deacetylases (HDACs). Site-specific reductions of acetylated histone H3 have been measured in clinical samples of prostate cancer in comparison to normal tissue, and in tumor cell lines, in parallel to increased HDAC activity [26]. Concordantly, another study reports significantly decreased histone H3 and H4 acetylation levels in prostate cancer [41]. On the other hand, high levels of global H3K18 acetylation are linked to a higher risk of recurrence [25], implying a deregulation of HATs and HDACs in prostate cancer.

Chromatin immunoprecipitation studies show that acetylated histone H3 peaks are found in the vicinity of AR binding regions and are characteristic of androgen-responsive genes [42]. Importantly, local hyper-acetylation and chromatin opening contribute to reduced androgen dependency in resistant prostate cancer models [43]. Recently, the role of hyper-acetylated super-enhancer regions as multi-molecular cooperative units that drive the expression of oncogenes has been outlined [44,45,46]. First studies related to the implication of such super-enhancers in prostate cancer are emerging and the enrichment of the acetyl mark binder bromodomain-containing protein 4 (BRD4) at genetic risk loci has recently been reported [47].

The AR interacts with numerous cofactors possessing HAT activity, which will impact the local histone acetylation status and downstream androgen-controlled gene expression [48]. In addition, several AR PTMs including lysine acetylation are probably catalyzed by the very same HATs [49,50,51]. Further, global expression profiling shows that EP300/KAT3B is an essential player involved in androgen target gene regulation [52]. It cooperates with GATA2 to open up chromatin at AR-targeted enhancers and facilitates gene expression [53]. The transcript levels of E1A-associated protein p300 (EP300) and the related cAMP-response element-binding protein (CREB) binding protein (CREBBP)/KAT3A are reduced by androgen but stimulated upon androgen ablation [54]. The dual, allosteric activator of EP300 and CREBBP I-CBP112 increases histone acetylation, mainly at H3K18, and impairs prostate cancer cell proliferation when applied at a low micromolar concentration [55]. MYST1/KAT8 also controls the activity of androgen target genes and its knockdown reduces prostate tumor cell proliferation [56,57]. TIP60/KAT5 is up-regulated in prostate cancer [58] and its impact on nuclear translocation following AR acetylation has been reported [59].

**Figure 1:**
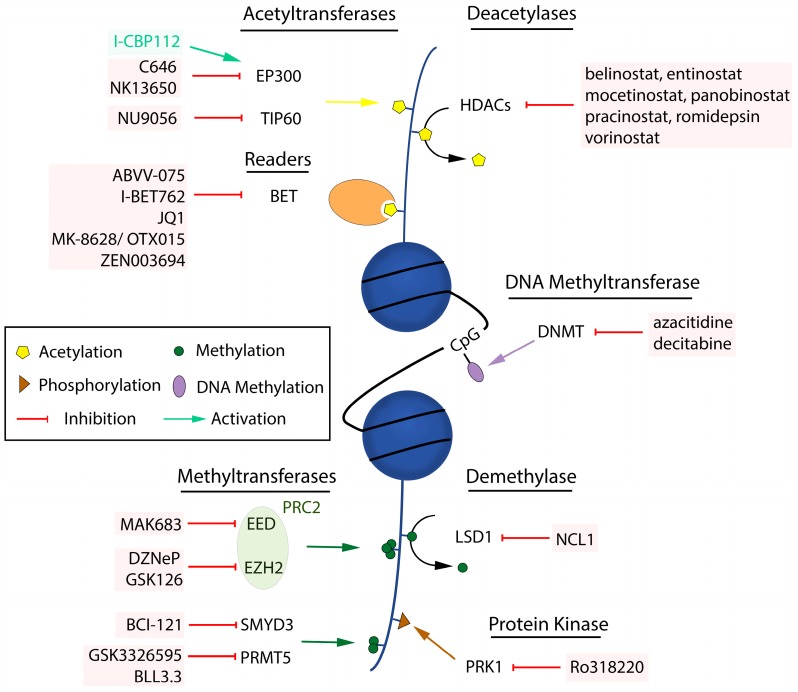
Overview of potential epigenetic targets and selected inhibitors. Straight arrows indicate the addition of acetyl (yellow), methyl (dark green) or phosphoryl (brown) groups to histones, or of a methyl group (purple) to DNA. Removal of these groups is indicated by half-circular black arrows.

Up to now, only few selective and potent HAT inhibitors are available. They address either the enzymatic activity or bromodomain function [60]. In some cases, their impact on prostate cancer models has been determined (Figure 1). The EP300 inhibitor C646 reduces AR function and induces apoptosis, but only at high doses [61]. The two related EP300 inhibitors NK13650A and NK13650B impair the viability of prostate cancer cells when given at high concentrations [62]. For TIP60 also, first inhibitory compounds have been identified [63]. Anti-proliferative effects and apoptosis induction were reported following in vitro treatment of prostate cancer cells with the TIP60 inhibitor NU9056 [64].

In line with site-specific reduction of histone acetylation marks, HDAC levels are elevated in prostate cancer, especially in high-grade tumors, and the levels of HDAC1 and HDAC2 are positively correlated with Gleason score [65,66]. The role of HDACs in androgen-driven gene expression via changes in local histone acetylation has been reported [67,68].

Inhibitors directed at the zinc-dependent HDAC family members have been around for many years (Figure 1), and their impact on the acetylation status of histone and non-histone proteins were described by numerous groups (see overview in [69]). Their efficacy was evidenced in several prostate tumor models. In vivo activity was, for instance, reported for the pan-HDAC inhibitors panobinostat and belinostat [70,71], and for the more selective inhibitors entinostat and mocetinostat [72,73]. Panobinostat was also shown to block growth of castration-resistant models [74]. Importantly, a stronger impact of HDAC inhibitors was observed in models harboring the *ERG* gene fusion, which is detected in about 50% of prostate tumors [75]. Concerning NAD^+^–dependent HDACs, it was described that sirtuin 1 directly interacts with the AR to locally reduce histone acetylation and repress its activity [76].

Bromodomain proteins are readers of histone acetylation marks that translate epigenetic modifications in their cellular context into a transcriptional response. The bromodomain and extra-terminal protein (BET) subgroup is probably the best studied one, due to the availability of highly potent and selective inhibitors (Figure 1) [77,78,79,80]. The role of BET proteins, mainly BRD4, in prostate cancer has been reported by several groups. Inhibitors of BET bromodomains with various chemical scaffolds such as JQ1, OTX015/MK-8628, I-BET762 or ABVV-075 exhibit strong anti-proliferative effects in different tumor xenografts, including models that respond poorly to anti-androgens [81,82,83,84,85]. A reduction of the expression and binding of AR full-length and of a splice variant found in resistant tumors was reported [84]. Another study shows that a model bearing an AR mutation responsible for enzalutamide resistance is still responsive to a combination treatment with JQ1 [86]. Also, BRD4 interacts with ERG to control the expression of common target genes which are up-regulated in CRPC. BET bromodomain inhibitors such as JQ1 and I-BET762 can partially prevent this interaction, implying an additional mechanism by which they reduce prostate tumor growth [87]. A newly described approach is the proteolysis targeting chimera (PROTAC) technology [88] where a BET bromodomain inhibitor linked to a ligand that recruits the E3 ubiquitin ligase von Hippel-Lindau was used for promoting degradation of the targeted BET proteins. A strong efficacy including tumor regression was observed in a CRPC model [89]. The marked effects observed for BET bromodomain inhibitors in several studies can be in part explained by the disruption of transcriptional networks as a consequence of the targeting of enhancers and super-enhancers which are required for proliferation and cellular identity [44].

Several other bromodomain proteins have been linked to prostate cancer. Examples include ATAD2, an AR cofactor up-regulated in a subset of prostate tumors [90], but no direct experiments probing its functional impact in vivo have been reported. Further, the transcriptional activator tripartite motif-containing 24 (TRIM24) is stabilized by speckle-type POZ protein (SPOP) mutations, which are often detected in recurrent prostate cancer [91,92]. TRIM24 and the AR have many common target genes and a direct cooperation between both regulators leading to enhanced downstream gene expression has been reported [92]. The levels of transcription initiation factor TFIID subunit 1 (TAF1), which is part of the basal multiprotein transcription complex TFIID, are linked with prostate cancer progression. This bromodomain protein stimulates the transcriptional activity of the AR, as shown by gene silencing experiments, probably by affecting AR ubiquitylation levels [93]. No data concerning the specific role of the respective bromodomains of TRIM24 or TAF1 are yet available, so that the recent discovery of inhibitors addressing these regions should greatly help to clarify this [94,95,96].

### 2.3. Histone Methylation

Dynamic changes in histone lysine methylation patterns during prostate cancer progression have been reported. For instance, elevated H3K4 dimethylation correlates with Gleason score and is associated with increased relapse risk [25,97]. Interestingly, the levels of this histone mark have recently been found to be stimulated by androgen treatment [98]. Also, H3K4 monomethylation, as well as H3K9 di- and trimethylation are diminished in prostate tumors compared to non-tumor tissues [41]. Another report shows that H4K20 methylation is much reduced in CRPC [99]. Far less is known about arginine methylation but H4R3 dimethylation is positively correlated with the Gleason score [97].

The AR interacts with several factors that govern histone methylation, most notably the polycomb repressive complex 2 (PRC2) [100]. A prominent member of this complex is an enhancer of zeste homolog 2 (EZH2), which is overexpressed in various cancer types, including prostate cancer where its elevated levels correlate with disease progression and higher Gleason score [101,102]. EZH2 catalyzes di- and trimethylation of H3K27, an essential mark associated with condensed and transcriptionally silent chromatin, thereby repressing gene transcription and disrupting differentiation processes, which may promote cancer stem cell development [103,104,105]. Beside its repressive role, EZH2 also acts independently of the PRC2 complex and co-activates gene transcription by interacting with transcription factors such as the AR [106], making it a promising target for prostate cancer treatment. Indeed, several recent in vitro and in vivo studies document that inhibition of EZH2 with compounds such as DZNeP and GSK126 alone (Figure 1), or in combination with other drugs, decreases prostate tumor size and proliferation [107,108]. Another essential component of the PRC2 complex is the embryonic ectoderm development protein (EED), for which selective inhibitors phenocopying EZH2 inhibitors have very recently been described [109,110,111,112]. EED inhibitors display potent in vitro and in vivo efficacy in different tumor types, also in models harboring an EZH2 mutation leading to resistance to inhibitors [109,110,111]. It will be interesting to find out whether tumors respond differently to inhibitors of EED or EZH2 inhibitors, and this will help to understand the respective roles of the H3K27me3 mark and of EZH2, as EED inhibition effectively reduces H3K27me3 levels but probably does not affect EZH2.

SET and MYND domain-containing protein 3 (SMYD3) methylates specific lysine residues in histones H3 and H4 but also in other proteins involved in cell proliferation pathways [113]. An elevated expression is predictive of prostate cancer aggressiveness and selective *SMYD3* gene silencing reduces tumor growth in vitro and in vivo [114,115]. A SMYD3 inhibitor named BCI-121 (Figure 1) with anti-proliferative effects on tumor cell lines including prostate cancer models has been described, and its activity was linked to SMYD3 levels [116].

Protein arginine methyltransferase 5 (PRMT5) has an oncogenic function in prostate tumor and other cancer types [117]. Apart from histones, it also methylates several additional proteins including the AR, thus regulating its activity on downstream target genes [118]. It furthermore controls AR levels upon interaction with the transcription factor Sp1 [119]. This was shown both by *PRMT5* knockdown studies and with the inhibitor BLL3.3 (Figure 1) which reduces *AR* gene transcription and H4R3 methylation [119].

Concerning histone demethylases (HDMs), overexpression has been observed for several of them in clinical prostate cancer samples. Examples include lysine-specific demethylase 1 (LSD1)/KDM1A, jumonji D2 (JMJD2)/JHDM3/KDM4, jumonji AT-rich interactive domain 1B (JARID1B)/KDM5B and PHD finger protein 8 (PHF8)/KDM7B [120,121,122,123,124]. A functional impact of HDMs on AR signaling has been reported by different groups and reviewed in detail [18,125]. This is the case for LSD1 which co-localizes with the AR and stimulates androgen-dependent gene transcription [126]. In addition, LSD1 cooperates with JMJD2C to control AR activity and regulates target gene expression via demethylation of H3K9 [127]. More recently it was found that LSD1 directly represses *AR* gene expression by removing H3K4 methylation marks in the second intron. This is not observed when androgen concentrations are low, which may explain the increased AR levels observed in patients relapsing during deprivation therapy [128]. NCL1 (Figure 1) is a recently described, low micromolar inhibitor of LSD1, which reduces growth of a CRPC model in vivo, while inducing apoptosis and autophagy [129]. Along with JMJD2C, JMJD2B also controls AR transcriptional activity as well as AR stability, by blocking its ubiquitylation [122]. PHF8 demethylates H4K20 and acts as an AR cofactor. Its expression is induced by hypoxia, which promotes late-stage prostate cancer progression, including neuroendocrine differentiation [130,131]. The ongoing efforts to identify better, highly potent and selective compounds that selectively inhibit individual HDMs will be of great help to further delineate the individual roles of members of this enzyme family in AR signaling and prostate cancer [132].

Chromodomain helicase DNA-binding protein 1 (CHD1) is a reader of H3K4 di- and trimethylation marks which is often mutated in *ETS* fusion-negative late-stage prostate cancer [133]. Its loss promotes prostate cancer aggressiveness [134,135] but also sensitizes tumor cells to inhibitors of the poly-ADP ribose polymerase, due to its role in the DNA damage response [136]. Interestingly, in PTEN-deficient prostate cancer, the inactivation of *CHD1* dramatically reduces proliferation and survival, due to its regulatory role on the tumor necrosis factor/nuclear factor kappa-light-chain-enhancer of activated B cells pathway [137].

### 2.4. Histone Ubiquitylation

Histone ubiquitylation is a chromatin mark associated mainly with the transcribed region of genes and involved in cellular differentiation as well as in DNA damage response [138,139,140]. The role of histone ubiquitylation in controlling AR function has only been analyzed in a few studies. The E3 ubiquitin ligases RNF20 and RNF40 stimulate H2B ubiquitylation and AR activity at target genes, and their depletion leads to impaired prostate cancer cell proliferation [141]. Ubiquitylation of the histone variant H2A.Z reduces AR activity and is controlled by USP10 [142]. Other studies show that direct ubiquitylation of the AR affects its stability and function [143,144,145,146]. Interestingly, the E3 ubiquitin ligase SPOP is a frequently inactivated tumor suppressor in prostate cancer [147]. It represses PI3K/mTOR signaling and its mutation promotes tumorigenesis, as evidenced in a mouse model [148]. The respective impacts of histone and AR ubiquitylation on downstream gene regulation remain to be exactly delineated [149,150].

### 2.5. Histone Phosphorylation

There are few published data on the impact of histone phosphorylation on AR signaling. H3T11 is phosphorylated by protein kinase N1 (PKN1), leading to androgen-mediated recruitment of the chromatin-associated protein WD repeat-containing protein 5 (WDR5) to AR target genes [151]. In line with this, inhibition of PKN1 with Ro318220 (Figure 1), and knockdown of *WDR5*, reduces androgen target gene expression and prostate cancer cell proliferation, respectively [151,152].

## 3. Clinical Studies in Prostate Cancer Addressing Epigenetic Targets

An overview of clinical trials performed with DNMT inhibitors and including prostate cancer patients is given in Table 1. Early studies with the demethylating compound decitabine showed only a limited efficacy in metastatic CRPC patients [153]. Presently, azacitidine is being evaluated in three clinical studies for prostate cancer treatment. In the most advanced one, it is tested in combination with docetaxel in chemotherapy-resistant metastatic CRPC patients to determine whether their response to docetaxel can be restored [154]. Phase 2 results show an objective response in three out of ten patients but more studies are needed to confirm this. Two additional trials are still ongoing but no concluding results are available yet.

Different HDAC inhibitors have been or are currently being assessed in clinical phase 1 or 2 for prostate cancer (Table 1). Early studies with vorinostat or romidepsin were disappointing as only limited responses were observed, probably due to the insufficient therapeutic window [155,156]. Similarly, a recently completed study with pracinostat showed only limited efficacy [157]. Panobinostat given as single agent leads to prostate-specific antigen decrease in a small number of patients only [158]. Combination treatments using different HDAC inhibitors and docetaxel or androgen deprivation are currently being evaluated but only few data are available until now [159,160]. To date, no phase 3 clinical trial has been performed, so the jury remains open as to whether HDAC inhibition is a valuable approach to treat prostate cancer patients [161].

BET bromodomain inhibitors with different chemical scaffolds are presently being tested in various tumor types, including prostate cancer in a few instances (Table 2). Two phase 1 trials focusing on metastatic CRPC evaluate ZEN003694 as single agent or in combination with the AR antagonist enzalutamide [162]. They started in 2016 and dose escalation is currently ongoing.

Several EZH2 inhibitors have entered clinical trials, but not for the indication of prostate cancer. However, the EED inhibitor MAK683 which targets the PRC2 complex just entered clinical phase 1 for different tumor types including prostate cancer (Table 2).

A clinical study with the PRMT5 inhibitor GSK3326595 (Figure 1) was recently initiated in patients with different malignancies, including prostate cancer (Table 2).

## 4. MicroRNAs as Potential Biomarkers for Prostate Cancer

Another way to take advantage of epigenetic alterations occurring in prostate cancer is to determine whether changes in microRNA profiles represent diagnosis biomarkers or are associated with natural disease progression and therapy response [163]. Following the discovery that miR-141 is elevated in prostate cancer and correlates with the serum PSA levels [164], a number of other microRNAs were found to be up-regulated (e.g., miR-20a, miR-21, miR-195 and miR-375) or down-regulated (e.g., miR-34a, miR-143/145, miR-205 and miR-488) in prostate cancer (for recent reviews see [28,165]. Importantly, several microRNAs including miR-34a and miR-34c directly control AR levels by targeting the 3′-untranslated region of the corresponding transcript [166]. This was also reported for miR-130b, which was furthermore shown to increase invasion and therapy resistance. In patients, miR-130b levels correlate with tumor stage and Gleason score [167]. On the other hand, the AR modulates the expression of some microRNAs which are involved in prostate cancer cell proliferation [28,165]. A few clinical trials are currently ongoing to evaluate circulating microRNAs as potential biomarkers for prostate cancer (Table 3). Their aim is either to identify risks for prostate cancer early on or to predict therapy response, but no final data are available yet.

## 5. Suppressor MicroRNAs as Potential Treatment for Prostate Cancer

Some preclinical studies suggest that treatment with suppressor microRNAs may represent a novel strategy for prostate cancer treatment, provided efficient delivery can be achieved. First experiments in which miR-15a, miR-16-1 [168], miR-34a [169], miR-124 [170] or miR-145 [171] were delivered into prostate tumor cells showed a reduction of proliferation. Importantly, intravenous miR-124 treatment of mice bearing a CWR22 xenograft results in significant tumor growth inhibition. This effect is further enhanced by additional enzalutamide treatment and linked to reduction of AR splice variant expression [170]. The levels of miR-455-3p are down-regulated in clinical prostate cancer samples and forced overexpression reduces prostate cancer cell growth in vivo [172]. Mechanistically, a reduction of cap-dependent translation due to destabilization of EIF4E transcripts by miR-455-3p has been evidenced [172].

Conversely, microRNAs with an oncogenic role in prostate cancer have also been described. One recent example is miR-4534 which controls the expression of PTEN. Reducing miR-4534 levels in a prostate tumor xenograft strongly impairs in vivo growth [173].

The respective impacts of these microRNAs on prostate cancer growth still need to be compared and more work will be necessary before the findings can be translated into the clinic. The miR-34a mimic MRX34 was evaluated in a clinical phase 1 study addressing solid tumors, but no mention was made of prostate cancer patients [174].

## 6. Conclusions and Perspectives

Complex epigenetic aberrations take place throughout the progression of tumors so that the determination of the global epigenetic landscape of prostate cancer will necessitate large cohorts of samples and concerted research efforts. Impressive progress has nonetheless already been achieved in the identification of epigenetic players involved in prostate cancer. Early findings on the role of DNA methylation and histone acetylation, and the subsequent discovery of bespoke DNMT and HDAC inhibitors ultimately led to extensive clinical testing. Unfortunately, this was not successful up to now, possibly due to the comparatively low proliferation rate of prostate tumor cells, especially in comparison to leukemias. Also, multiple side-effects leading to lack of therapeutic window have been reported in many instances, implying that more selective drugs and stratification strategies to identify prostate cancer subgroups will be essential in defining the patient subpopulation most likely to respond to such treatments. Concerning novel epigenetic targets such as BET proteins, we should soon know whether their specific inhibitors are successful in the clinical setting. Ultimately, combination with an additional agent may prove more beneficial for increased efficacy and also to delay therapy resistance. In this line it will be interesting to find out whether immune checkpoint inhibitors can be successfully combined with epigenetic drugs, especially in the area of prostate cancer where responses have been limited so far [175]. Altogether, the tremendous progress recently made in large-scale analyses of genomic and epigenetic alterations will provide invaluable information for the identification of novel targets, development of novel therapies and stratification of patients.

## Figures and Tables

**Table 1:** Clinical studies in prostate cancer, including castration-resistant prostate cancer (CRPC) and metastatic CRPC (mCRPC), with drugs addressing DNA methyltransferases (DNMTs) or histone deacetylases (HDACs). Source: https://clinicaltrials.gov/.

Target	Compound	Combination	Indication	Phase	Identifier	Status
DNMT	Azacitidine	Phenylbutirate	Prostate cancer	2	NCT00006019	Completed
DNMT	Azacitidine		Prostate cancer	2	NCT00384839	Completed
DNMT	Azacitidine	Docetaxel, prednisone	mCRPC Post-chemotherapy	1/2	NCT00503984	Terminated
HDAC	Vorinostat		Includes prostate cancer	1	NCT00005634	Completed
HDAC	Vorinostat		Includes prostate cancer	1	NCT00045006	Completed
HDAC	Vorinostat		Advanced CRPC Post-chemotherapy	2	NCT00330161	Completed
HDAC	Vorinostat	Docetaxel	Includes prostate cancer	1	NCT00565227	Terminated
HDAC	Vorinostat	Androgen deprivation	Localized prostate cancer	2	NCT00589472	Completed
HDAC	Vorinostat	Temsirolimus	mCRPC	1	NCT01174199	Terminated
HDAC	Entinostat		Includes prostate cancer	1	NCT00020579	Completed
HDAC	Romidpesin		Prostatic neoplasms	2	NCT00106301	Completed
HDAC	Romidepsin		mCRPC	2	NCT00106418	Completed
HDAC	Romidepsin		CRPC	1	NCT01638533	Recruiting
HDAC	Belinostat		Includes prostate cancer	1	NCT00413075	Completed
HDAC	Belinostat	5-fluorouracil	Includes prostate cancer	1	NCT00413322	Completed
HDAC	Panobinostat	Docetaxel, prednisone	CRPC	1	NCT00419536	Terminated
HDAC	Panobinostat	Docetaxel, prednisone	CRPC	1	NCT00493766	Terminated
HDAC	Panobinostat	Docetaxel, prednisone	CRPC	1	NCT00663832	Completed
HDAC	Panobinostat		mCRPC	2	NCT00667862	Completed
HDAC	Panobinostat	External beam radiotherapy	Includes prostate cancer	1	NCT00670553	Completed
HDAC	Panobinostat	Bicalutamide	Recurrent CRPC	1/2	NCT00878436	Completed
HDAC	Mocetinostat	Docetaxel	Includes prostate cancer	1	NCT00511576	Terminated
HDAC	Valproic acid		CRPC	2	NCT00670046	Unknown
HDAC	Pracinostat		mCRPC	2	NCT01075308	Completed

**Table 2:** Clinical studies in prostate cancer with drugs addressing the novel epigenetic targets bromodomain and extra-terminal protein (BET), embryonic ectoderm development protein (EED) and protein arginine methyltransferase 5 (PRMT5). Source: https://clinicaltrials.gov/.

Target	Compound	Combination	Indication	Phase	Identifier	Status
BET	GSK525762		CRPC	1	NCT01587703	Recruiting
BET	OTX105 MK-8628		CRPC	1	NCT02259114	Active, not recruting
BET	OTX105 MK-8628		CRPC	1	NCT02698176	Active, not recruting
BET	INCB054329		CRPC	1/2	NCT02431260	Recruiting
BET	INCB057643		CRPC	1/2	NCT02711137	Recruitng
BET	ZEN003694		mCRPC	1	NCT02705469	Recuiting
BET	ZEN003694	Enzalutamide	mCRPC	1b	NCT02711956	Recruiting
EED	MAK683		Includes prostate cancer	1	NCT02900651	Recruiting
PRMT5	GSK3326595		Includes prostate cancer	1	NCT02783300	Recruiting

**Table 3:** Clinical studies in prostate cancer, including metastatic castration-resistant prostate cancer (mCRPC), evaluating microRNAs (miRNA) as potential biomarkers. Source: https://clinicaltrials.gov/.

Outcome Measure	Treatment	Indication	Identifier	Status
miRNA profiling		Prostate cancer	NCT01220427	Terminated
miRNA profiling using Nano-string technology		Prostate cancer	NCT02964351	Not yet recruiting
Serum exosomal miRNA profiling using next-generation sequencing	Androgen deprivation	Prostate cancer	NCT02366494	Recruiting
Preselected miRNA profiling	Enzalutamide	mCRPC	NCT02471469	Recruiting
miRNA profiling	Radiotherapy	Prostate cancer	NCT02745587	Recruiting
Preselected miRNA profiling	Abiraterone acetate	mCRPC	NCT01503229	Ongoing, not recruiting
miRNA-141, -375 levels	Focal brachytherapy	Low-risk prostate cancer	NCT02391051	Recruiting
miRNA profiling	Androgen deprivation + cixutumumab	Metastatic prostate cancer	NCT01120236	Ongoing, not recruiting
